# Protective Effect Against Toxoplasmosis in BALB/c Mice Vaccinated With *Toxoplasma gondii* Macrophage Migration Inhibitory Factor

**DOI:** 10.3389/fmicb.2019.00813

**Published:** 2019-04-24

**Authors:** Kang Liu, Hongyang Wen, Haijian Cai, Minmin Wu, Ran An, Deyong Chu, Li Yu, Jilong Shen, Lijian Chen, Jian Du

**Affiliations:** ^1^Department of Biochemistry and Molecular Biology, School of Basic Medical Sciences, Anhui Medical University, Hefei, China; ^2^Anhui Provincial Key Laboratory of Microbiology and Parasitology, Anhui Medical University, Hefei, China; ^3^Department of Anesthesiology, The First Affiliated Hospital of Anhui Medical University, Hefei, China

**Keywords:** *T. gondii*, toxoplasmosis, TgMIF, protein vaccine, protective efficacy

## Abstract

*Toxoplasma gondii* is an obligate intracellular parasite responsible for toxoplasmosis, which can cause severe disease in the fetus and immunocompromised individuals. Developing an effective vaccine is crucial to control this disease. Macrophage migration inhibitory factor (MIF) has gained substantial attention as a pivotal upstream cytokine to mediate innate and adaptive immune responses. Homologs of MIF have been discovered in many parasitic species, and one homolog of MIF has been isolated from the parasite *Toxoplasma gondii.* In this study, the recombinant *Toxoplasma gondii* MIF (rTgMIF) as a protein vaccine was expressed and evaluated by intramuscular injection in BALB/c mice. We divided the mice into different dose groups of vaccines, and all immunizations with purified rTgMIF protein were performed at 0, 2, and 4 weeks. The protective efficacy of vaccination was analyzed by antibody assays, cytokine measurements and lymphoproliferative assays, respectively. The results obtained indicated that the rTgMIF vaccine elicited strong humoral and cellular immune responses with high levels of IgG antibody and IFN-γ production compared to those of the controls, in addition to slight higher levels of IL-4 production. After vaccination, a stronger lymphoproliferative response was also noted. Additionally, the survival time of mice immunized with rTgMIF was longer than that of the mice in control groups after challenge infection with virulent *T. gondii* RH tachyzoites. Moreover, the number of brain tissue cysts in vaccinated mice was reduced by 62.26% compared with the control group. These findings demonstrated that recombinant TgMIF protein is a potential candidate for vaccine development against toxoplasmosis.

## Introduction

*Toxoplasma gondii* is an obligate intracellular protozoan that belongs to the phylum Apicomplexa. This parasite can infect a wide range of warm-blooded mammals, including humans, livestock and birds. According to the statistics, approximately one-third of the world’s population is seropositive (Petersen et al., 2001; Munoz et al., 2011). The parasite causes severe symptoms in immunocompromised patients, as well as abortions in infected pregnant women (Martin et al., 2004; Ching et al., 2016). Moreover, the obtained results from the epidemiologic survey show that the prevalent disease of *T. gondii* has caused large economic losses to the stock-raising industry (Dubey et al., 2005; Wang et al., 2016).

Currently, medications (pyrimethamine, sulfadiazine, and spiramycin) can control *T. gondii* acute infections, but they are unable to eliminate the chronic infection completely (Montoya and Liesenfeld, 2004; Innes, 2010). Vaccinations are considered to be an effective mean to prevent infection with the parasite. To date, inactivated and attenuated vaccines, subunit vaccines, DNA vaccines and recombinant protein vaccines have been developed to prevent infection by *T. gondii*. However, inactivated and attenuated vaccines are not sufficiently efficacious against this ubiquitous pathogen. In addition, it has been recognized that DNA vaccines generate only weak immune responses when used in higher primates and humans (Kur et al., 2009; Liu et al., 2012). Toxovax^®^, the only available commercial vaccine, is based on the live-attenuated *T. gondii* S48 strain and is licensed only for use in sheep to avoid congenital toxoplasmosis (Buxton and Innes, 1995). However, due to the risk of reverting to a virulent wild type, live vaccines need to be further explored in food-producing animals and humans. Thus, it is imperative for us to develop a valuable and practical vaccine against toxoplasmosis, and an effective vaccine will be beneficial to the livestock industry by reducing economic losses (Qu et al., 2013).

Macrophage migration inhibitory factor (MIF) is a pro-inflammatory mammalian cytokine with enzymatic activity of tautomerase and oxidoreductase. MIF has been demonstrated to be involved in innate and adaptive immune responses by stimulating the production of pro-inflammatory mediators, such as TNF-α and IL-8. MIF was found to sustain macrophage survival and proinflammatory function by suppressing activation-induced, p53-dependent apoptosis (Mitchell et al., 2002; Calandra and Roger, 2003; Larson and Horak, 2006; Hertelendy et al., 2018). The cytokines affected by MIF are involved in the pathogenesis of infectious and non-infectious inflammatory diseases (de Oliveira Gomes et al., 2011; Gomes et al., 2018). In a previous study, this conserved protein homolog has been found in different phyla, including many parasitic organisms, and it was capable of facilitating the host immune responses during infection (Zang et al., 2002; Flores et al., 2008; Vermeire et al., 2008; Sommerville et al., 2013; Sparkes et al., 2017). Considering the important roles of MIF, we hypothesized that the homolog of MIF from *Toxoplasma gondii* (TgMIF) could be utilized as a candidate vaccine against *Toxoplasma* infection.

The aim of this study was evaluating the mouse immunoreaction after injecting the rTgMIF protein vaccine and examining the efficacy of TgMIF as a vaccine candidate against acute and chronic *T. gondii* infection.

## Materials and Methods

### Ethics Statement

Ethical permission was obtained from the Institutional Review Board of the Institute of Biomedicine at Anhui Medical University (permit number 20180016), which records and regulates all research activities in the school. This study was carried out in strict accordance with the recommendations of the Guide for the Care and Use of Laboratory Animals of Anhui Medical University. The experiments were subjected to approval by the Ethics Committee of the Animal Experiments of Anhui Medical University. The approval from the Institutional Review Board includes permission for using mice under anesthesia, and all of the experimental procedures were performed in strict accordance with the recommendations in the Guide for the Care and Use of Laboratory Animals of the National Institutes of Health.

### Animals and Parasites

Six- to eight-week-old female BALB/c mice were purchased from the Anhui Medical University Laboratory Animal Center. All mice were bred under specific-pathogen-free conditions and had free access to food and tap water. The virulent wild-type RH strain (Type 1) and PRU strain (Type 2) were propagated in our laboratory. The tachyzoites of RH were harvested from human foreskin fibroblast cells. The PRU strain of *T. gondii* used in this study was maintained by passage of cysts in Kunming mice.

### TgMIF Antibody Preparation

Specific TgMIF antibody was obtained by subcutaneous immunizations of a rabbit with 1 mg of the recombinant protein separated on sodium dodecyl sulfate polyacrylamide gel electrophoresis (SDS–PAGE) and transferred to nitrocellulose, which was then crushed in Freund’s complete adjuvant and injected. Two further injections were performed at 3-week intervals in Freund’s incomplete adjuvant. The rabbit was then bled, and the specific antibodies were affinity purified on the recombinant protein that was electrophoresed and transferred onto nitrocellulose for subsequent Western blot analysis.

### Expression and Purification of Recombinant TgMIF and Tg14-3-3

Amplification of the open reading frame encoding *T. gondii* MIF (GenBankTM ID XP_002368429.1) was achieved through RT-PCR of the whole *T. gondii* (ME49 strain) RNA. The gene sequence encoding TgMIF was inserted into the prokaryotic expression vector pET28a (Novagen). Amplification of the open reading frame encoding *T. gondii* 14-3-3 (GenBankTM ID AB012775) was achieved through RT-PCR of the whole *T. gondii* (RH strain) RNA. The gene sequence encoded Tg14-3-3 was inserted into the prokaryotic expression vector pET28a (Novagen). To express the recombinant protein, the pET-28a-TgMIF or pET-28a-Tg14-3-3 plasmid was transformed into *Rosetta* (DE3) host bacteria cells (Transgen, CN), and recombinant protein expression was induced with 0.1 mM IPTG at 30°C for 4 h with constant shaking at 220 rpm. Then, the bacterial cells were harvested by centrifugation at 12,000 rpm for 5 min at 4°C. The obtained pellets were resuspended in lysis buffer (50 mM NaH_2_PO_4_, 300 mM NaCl, 10 mM imidazole, pH 8.0), the bacteria suspension was sonicated at a speed of 300 W/s on ice, and the total ultrasonic time was 10 min at 5 s interval. In the next step, the lysate was centrifuged at 12,000 × *g* for 20 min to separate the supernatant and bacteria debris. The level of rTgMIF expression was analyzed by 15% SDS–PAGE electrophoresis and Coomassie blue R-250 staining.

The subsequent purification steps were implemented at 4°C. The separated supernatants were collected and mixed with a purification column containing 1 ml Ni^2+^-NTA agarose (Qiagen, Germany). The complex was incubated for 1 h with gentle rotating. After binding, the binding protein was washed with wash buffer 1 (50 mM NaH_2_PO_4_, 300 mM NaCl, 20 mM imidazole, pH 8.0) and wash buffer 2 (50 mM NaH_2_PO_4_, 300 mM NaCl, 40 mM imidazole, pH8.0) for removing other unrelated proteins. Subsequently, the target protein was eluted with elution buffer (50 mM NaH_2_PO_4_, 300 mM NaCl, 300 mM imidazole, pH 8.0). The eluted rTgMIF protein was collected and dialyzed overnight against PBS to remove unwanted salt ions. The efficiency of purification was analyzed via 15% SDS–PAGE.

### Western Blotting

The rTgMIF and rTg14-3-3 were prepared as described above. The supernatants were separated on 15% SDS–PAGE gels and then electrophoretically transferred to PVDF membranes (GE Healthcare). The membranes were blocked in 5% skim milk for 1 h at room temperature and then incubated at 4°C overnight with the rabbit anti-TgMIF primary antibody (1:1000). The PVDF membrane was subsequently incubated with anti-rabbit HRP-IgG (Beyotime, China) for 1 h at room temperature, and chemiluminescence was detected using an ECL blot detection system (Engreen, China).

### rTgMIF Immunization

Initially, 6- to 8-week-old female BALB/c mice (*n* = 240) were divided into six groups with 40 mice per group at random. We set two negative groups: a blank control and PBS, and four immunization groups (protein doses of 2.5, 5.0, 10.0, or 20.0 μg). The doses of immunization were referenced according to previously studies (Pinzan et al., 2015; Ching et al., 2016; Sonaimuthu et al., 2016; Czarnewski et al., 2017). For the experimental group, all mice in different groups were intramuscularly injected with 2.5, 5.0, 10.0, or 20.0 μg of rTgMIF emulsified in Freund’s adjuvant, respectively. As negative controls, the mice were intramuscularly injected with 100 μl sterile PBS added with adjuvant. Different immunization samples were emulsified with complete/incomplete Freud’s adjuvant (C/IFA) at a 1:1 ratio before immunization. The first immunization was with Freund’s complete adjuvant, and the next two immunizations were with Freund’s incomplete adjuvant for booster injections. Mice were immunized using the same protocol on days 1, 15, and 29. Blood serum samples were collected from the immunized mice in each group via tail-bleeding on days 0, 14, 28, and 42. Sera samples were separated and stored at -80°C until evaluation of the levels of specific antibodies.

### Antibody Detection

Serum samples were harvested on days 0, 14, 28, and 42 and stored at -80°C. The level of antigen-specific IgG was evaluated by enzyme-linked immunosorbent assays (ELISAs). In brief, polystyrene 96-well flat-bottom microtiter plates were coated with 10 μg/mL rTgMIF diluted in 100 μl coating buffer (50 mM carbonate buffer, pH 9.6) at 37°C for 1 h, and then incubated overnight at 4°C. The plates were washed five times with PBS containing 0.05% Tween20 (PBST, pH7.4) and then we blocked the plates with 1% BSA for 1 h at 37°C. Thereafter, after washing five times with PBST, they were incubated with sera diluted in 0.1% BSA-PBST (1:50 for IgG) for 1 h at 37°C. After washing, 100 μl of HRP-conjugated goat anti-mouse IgG (ProteinTech Group, Inc., United States) diluted in PBST (1:1000) was added to the wells and incubated for 1 h at 37°C. Then, the plates were washed, and the immune complexes were represented by incubating with TMB (100 μl/well, Beyotime Biotechnology) for 20 min. The enzyme reaction was terminated by adding 2 M H_2_SO_4_. The optical density (OD) was measured with an ELISA reader at 450 nm. All sera samples were measured in triplicate.

### Lymphocyte Proliferation Assays

Two weeks after the last immunization, three mice per group were anesthetized and sacrificed. The spleens were harvested under aseptic conditions in PBS and passed through a 70 μm wire mesh sieve to generate a single cell suspension. The splenic lymphocytes were disposed of using standard lymphocyte isolation lysis buffer. Splenocytes were cultured in 96-well microplates at a density of 2 × 10^5^ cells per well in RPMI-1640 containing 10% FBS. The spleen cells were stimulated with 10 μg/mL rTgMIF (positive control) or medium alone (negative control) for 72 h at 37°C in a 5% CO_2_ incubator. Splenocytes proliferation assay was conducted by using a Cell Counting Kit-8 (Beyotime Biotechnology, CN). The stimulation index (SI) was expressed using the formula OD_450_ rTgMIF/OD_450_M, which was the ratio of the OD_450_ of stimulated cells to the OD_450_ of unstimulated cells.

### Cytokine Assays

To detect the levels of cytokine production, spleen cells were obtained from three mice in each group as described. For these experiments, 1.5 × 10^6^ cells/well were seeded into 24-well plates and stimulated with 10 μg/mL rTgMIF. Cell-free supernatants were harvested to measure IL-4 activity at 24 and 96 h for quantification of IFN-γ after poststimulation, respectively. All assays were performed with commercial ELISA kits according to the manufacturer’s instructions (Shanghai Enzyme-linked Biotechnology Co., Ltd., China). The levels of IFN-γ and IL-4 were determined by reference to standard curves constructed with known amounts of mouse recombinant IFN-γ and IL-4. The analysis was performed with data from three independent experiments.

### Challenge Infection

The best immune group was selected to infect with *T. gondii*. A total of 30 mice per group were randomly chosen and challenged with 1 × 10^3^ *T. gondii* RH strain tachyzoites diluted in 100 μl PBS by intraperitoneal injection 2 weeks after the final immunization. The survival time of the challenged mice was recorded within 30 days, and their survival rate was calculated until there was a fatal outcome for all animals. To evaluate the protection efficiency of the vaccine against chronic toxoplasmosis, three mice per group were orally challenged with 20 tissue cysts of the PRU strain as a model of experimental chronic toxoplasmosis. One month after infection, brains of mice from each group were homogenized in 1 ml PBS. The number of cysts per brain was determined by three samples of 10 μl aliquots of each homogenized brain under an optical microscope.

### Statistical Analysis

Statistical analysis and graphics were performed following the procedures of GraphPad Prism 5.0. All data, including antibody levels and lymphocyte proliferation assays, cytokine production and brain cyst loadings were compared by one-way ANOVA. The survival time was evaluated by the Kaplan-Meier method and compared with the log-rank test. The value of *p* < 0.05 was considered to be statistically significant.

## Results

### Epitope Analysis

**Figure 1:**
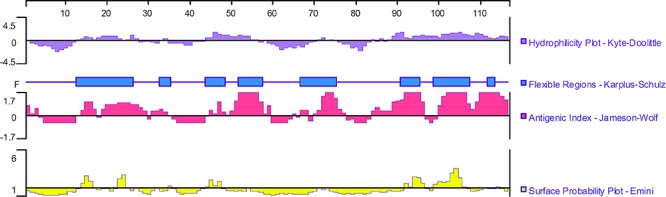
Linear-B cell epitopes of TgMIF predicted by DNASTAR in hydrophilicity plot, flexible regions, antigenic index, and surface probability rules.

The ORF of TgMIF is 351 bp, encoding a protein of 116 amino acids with a predicted molecular weight of 13 kDa and an isoelectric point of 8.91. DNASTAR was used to analyze the protein of TgMIF for surface probability, antigenic index, and hydrophilic plot, as well as flexible region. The results revealed that most regions of the TgMIF protein were in hydrophilicity plots and flexible regions, and TgMIF exhibited ideal surface probability and an antigenic index that indicated it was a promising prospect for producing vaccines (Figure 1).

### Recombinant TgMIF Expression and Purification

**Figure 2:**
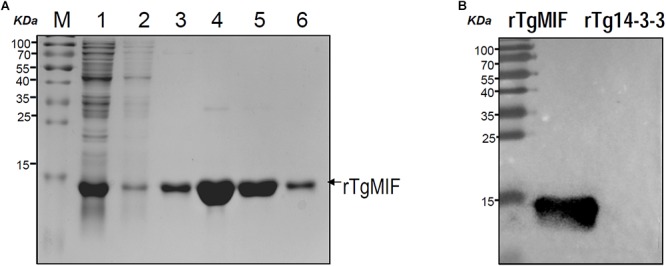
SDS-PAGE and western blotting analysis of the purified rTgMIF protein. **(A)** rTgMIF protein was purified and stained with Coomassie blue. The protein band of rTgMIF was approximately 13 kDa. M: protein marker; Lane 1: total bacterial lysate of pET-28a-TgMIF; Lanes 2–3: wash 1 and wash 2, the purified protein was washed, and the washing product was detected; Lanes 4–6: elution product, the purified rTgMIF was eluted three times, Elution 1, Elution 2, and Elution 3. **(B)** The purified rTgMIF and rTg14-3-3 were detected through western blotting by anti-TgMIF polyclonal antibody. M: protein molecular weight marker; rTgMIF and rTg14-3-3 purified through Ni^2+^-charged column chromatography and after dialysis.

The recombinant TgMIF was expressed in *E. coli* and the soluble recombinant protein was purified by Ni^2+^-NTA agarose. SDS-PAGE analysis indicated that the rTgMIF had a molecular weight of approximately 13 kDa (Figure 2A). In addition, the specificity of prokaryotic expression of rTgMIF was identified by the polyclonal antibody against TgMIF as a band of approximately 13 kDa, which was consistent with the deduced size. In addition, another purified recombinant parasite protein, Tg14-3-3, was not identified by the TgMIF antibody (Figure 2B). Thus, these results indicated that rTgMIF had been successfully expressed and purified.

### Humoral Immune Response Elicited by the Immunized Mice

**Figure 3:**
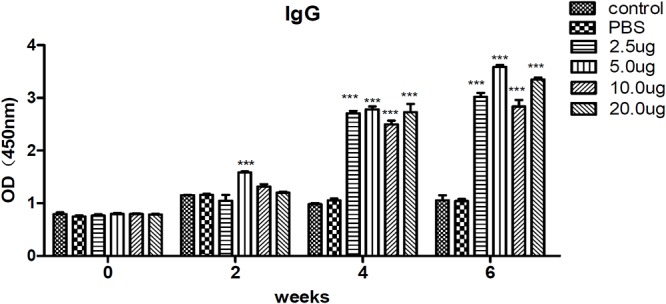
Detection of specific IgG antibodies in mice sera. IgG antibodies were determined and evaluated by ELISA. Serum samples of each group were collected and detected on the preinjection, second, fourth, and sixth week after immunization. The results are expressed as the mean ± SD (*n* = 3). Statistical differences are indicated by ^∗∗∗^ (highly significant; *p* < 0.001) compared with the PBS group.

To evaluate the levels of specific anti-*T. gondii* antibodies in sera, IgG from different groups were tested by ELISA on the preinjection, second, fourth, and sixth week after inoculation. The results revealed that IgG remained stable, and there was no difference in the blank control and PBS groups. However, the levels of IgG were significantly increased in the sera of mice immunized with rTgMIF compared with the control groups (*p* < 0.001). Moreover, the level of IgG tended to increase along with the continuous injection. The results also indicated that 5 μg rTgMIF induced a humoral immune response in the second week, and other doses induced a response in the fourth week. Moreover, a dose of 5 μg rTgMIF elicited the maximum level of IgG (Figure 3).

### Evaluation of Lymphocyte Proliferation

**Figure 4:**
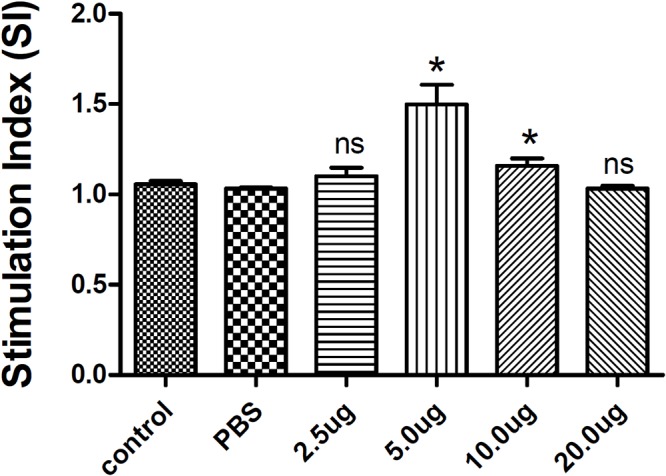
Splenocyte proliferation assay in mice. Two weeks after the last immunization, spleen lymphocytes were collected from vaccinated mice. The proliferative response was evaluated by CCK-8 assay. The results are expressed as the stimulation index (SI) ± SD (*n* = 3). Statistical differences are represented by ^∗^(*p* < 0.05) in comparison with the PBS control group. ns: no significant.

Splenocytes from each group were cultured to evaluate the antigen-specific lymphocyte proliferative response by CCK-8 assay. As expected, there was no significant difference between the two control groups (the blank and PBS control groups). However, lymphocyte proliferation *in vitro* assays revealed that splenic lymphocytes from mice immunized with 5 μg or 10 μg rTgMIF had a significantly higher proliferative response compared with the blank or PBS control groups (Figure 4). These results suggested that immunization with rTgMIF induced antigen-specific lymphocytes in mice.

### Expression of Cytokines

**Figure 5:**
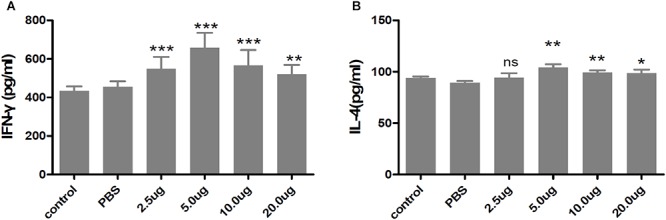
Cytokines secreted by splenocytes of mice immunized with rTgMIF protein. **(A)** IFN-γ. **(B)** IL-4. Each bar represents the mean ± SD (*n* = 9). ^∗∗∗^*p* < 0.001, ^∗∗^*p* < 0.01, ^∗^*p* < 0.05, ns: no significant.

The cell-mediated immunity induced in the immunized mice was evaluated by measuring the levels of cytokines released in the supernatants of cultures of rTgMIF-stimulated spleen cells. The levels of IFN-γ and IL-4 secreted from the spleen cells of all of the rTgMIF-immunized mice were increased compared with the PBS group, and 5 μg of rTgMIF elicited cell-mediated immunity more efficiently compared to the other dosages (Figure 5). It is known that IFN-γ favors Th1-type immune responses, whereas IL-4 is correlated with a Th2-type response. The rTgMIF vaccine induced high levels of IFN-γ and slight higher low IL-4 levels, indicating that a bias toward the Th1-type immune response was induced by rTgMIF vaccines. Therefore, these data indicated that the cellular immune response induced by rTgMIF was oriented toward a Th1 profile in the immunized mice.

### Evaluation of Protective Activity in BALB/c Mice

**Figure 6:**
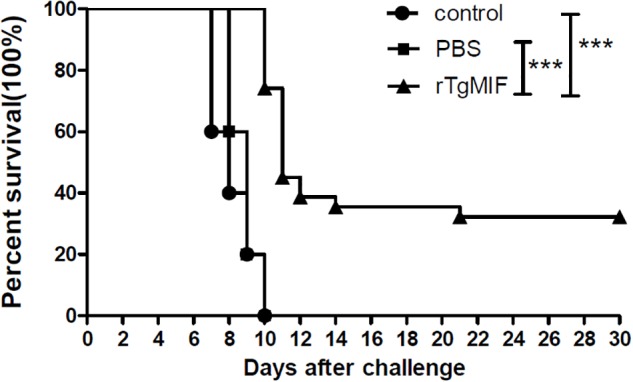
Survival rates of the immunized BALB/c mice. Three groups of mice (*n* = 30) were challenged with 1 × 10^3^ tachyzoites of *T. gondii* virulent RH strain intraperitoneally 2 weeks after the last immunization. Mice immunized with rTgMIF exhibited a significant increase in the survival days (median survival of 11 days) in comparison to the blank control or PBS group (median survival of 8 and 9 days, respectively). ^∗∗∗^*p* < 0.001, Gehan–Breslow–Wilcoxon tests.

To evaluate whether the rTgMIF protein could confer effective protection against acute toxoplasmosis, different groups were injected with 1 × 10^3^ tachyzoites of the virulent RH strain intraperitoneally. The survival curves of mice from different groups are shown in Figure 6. Mice in the groups of blank control and PBS died from 7 to 10 days after the challenge, and there were no survivors in either group. The two groups showed no significant difference in survival time (median survival of 8 and 9 days, respectively). It was revealed that the mice immunized with 5 μg rTgMIF (median survival of 11 days) had a significantly prolonged survival rate in comparison to the two control groups (blank control or PBS) (*p* < 0.0001, respectively). Surprisingly, 25% of the immunized mice survived to the 30th day. These results demonstrated that rTgMIF conferred some degree of protection against *T. gondii* acute infection in mice.

**Figure 7:**
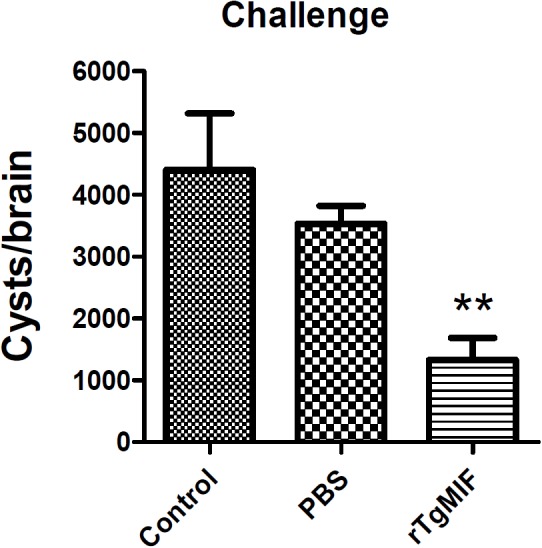
Total brain cysts in mice after challenge infection. Mice were orally challenged with 20 tissue cysts of the *T. gondii* PRU strain 2 weeks after the last immunization. The number of brain cysts was counted, and the results are expressed as the mean ± SD (*n* = 3). Statistical differences are represented by ^∗∗^ (*p* < 0.01).

Furthermore, the protein vaccine was evaluated against *T. gondii* chronic infection. Mice were orally challenged with 20 cysts of the *T. gondii* PRU strain. One month after infection, the number of brain cysts was counted. As shown in Figure 7, the number of cysts in mice immunized with rTgMIF (1333.33 ± 352.8) was significantly lower compared with the groups of blank control (4400 ± 916.5) and PBS (3533.33 ± 290.6) (*p* < 0.01). The number of brain cysts in vaccinated mice showed a 62.26% reduction compared with the PBS group. In addition, the size of the brain cysts in vaccinated mice was obviously smaller than that in the two control groups (data not shown). These results revealed that rTgMIF protein enhanced the immunity of mice against *T. gondii* chronic challenge.

## Discussion

*Toxoplasma gondii* is a ubiquitous intracellular apicomplexan parasite that infects many species of animals from diverse geographic regions; thus, it is necessary to develop effective vaccines to prevent and control the spread of toxoplasmosis. The only commercially live attenuated vaccine, Toxovax^®^, derived from the attenuated tachyzoites of the *T. gondii* S48 strain, has been used to reduce abortion in sheep (Buxton and Innes, 1995). In addition, genetically modified parasites, such as the uracil auxotroph mutant, also have yielded promising results in the experimental setting (Fox and Bzik, 2002, 2010, 2015; Xia et al., 2018). However, these vaccines may revert to a pathogenic strain and therefore are not suitable for human use. To date, considerable research has been conducted on DNA vaccines or DNA-cocktail vaccines, with few protein vaccines being tested (Yan et al., 2012; Chen et al., 2013, 2015; Hassan et al., 2014; Zhang et al., 2015; Zhang N.Z. et al., 2018; Zhang Z. et al., 2018). It is well-known that protein vaccines elicit strong humoral responses by inducing antigen-specific antibodies. In this work, a recombinant protein vaccine containing the ORF of TgMIF was prepared.

Migration inhibitory factor is an ancient pro-inflammatory mammalian cytokine with multiple functions, such as pro-inflammatory, chemotactic and growth-promoting activities. In addition, MIF has gained substantial attention as a pivotal upstream mediator of innate and adaptive immune responses. The proinflammatory activity of MIF has been implicated in a diverse range of biological processes including activation of ERK/MAPK pathways and inhibition of the anti-inflammatory actions of glucocorticoids. MIF has also been shown to induce the production of downstream pro-inflammatory gene expression, such as TNF-α, IL-8, and IL-17 (Calandra and Roger, 2003; Larson and Horak, 2006; Hertelendy et al., 2018). MIF plays an important role in host defense against a variety of microorganisms including protozoan parasites (Satoskar et al., 2001; Calandra and Roger, 2003; Magalhaes et al., 2009; Cavalcanti et al., 2011). Previous study revealed that MIF is essential for controlling parasites burden with increasing inflammatory response and tissue damage after oral infection with *T. gondii* (Cavalcanti et al., 2011). Additionally, it is revealed that that MIF-induced early dendritic cell maturation and IL-12 production mediate resistance to *T. gondii* infection (Terrazas et al., 2010). Recent study suggested that the host NADPH oxidase 4 is required for the generation of MIF and host defense against *T. gondii* infection (Kim et al., 2017). Therefore, the host MIF participates in anti-*T. gondii* defense. Interestingly, some microbial pathogens also express a MIF-like protein. Homologs of MIF have been discovered in many parasitic species infecting mammalian hosts and it has been suggested that parasites express MIF to manipulate the host immune response during infection (Zang et al., 2002; Flores et al., 2008; Vermeire et al., 2008; Sparkes et al., 2017). However, not surprisingly, MIF encoded by the hosts and microbial pathogens display distinct immunoregulatory properties. For example, Chicken macrophages treated with chicken MIF (C.MIF) protein expressed increased levels of IL-6, IL-17, and decreased level of IL-8, whereas Eimeria-encoded MIF (E.MIF) treatment only down-regulated IL-8. Moreover, E.MIF, but not C.MIF, enhanced protection against experimental Eimeria infection (Jang et al., 2011). Interspecies sequence alignments revealed that TgMIF has 26% identity with mammalian MIFs from host species (Sommerville et al., 2013). In this study, the protein sequence of TgMIF was analyzed by DNASTAR. The results suggested that most regions of the TgMIF protein were in hydrophilicity plots and flexible regions, and TgMIF had an excellent antigenic index and surface probability, indicating the positive antigenicity of TgMIF. Moreover, TgMIF shows 99.7% nucleotide sequence identity and 100% identity of amino acids from all three strains, indicating it can be considered as a vaccine candidate against various strains of *T. gondii.* Recently, Plasmodium-encoded MIF ortholog was found to be a good vaccine conferring complete protection against malaria re-infection (Baeza Garcia et al., 2018). Therefore, we speculate that MIF ortholog from *T. gondii* may be an ideal material for producing an anti-*T. gondii* protein vaccine.

In our study, 25% of immunized mice survived to the 30th day after challenge infection with type 1 RH tachyzoites, whereas there were no survivors in the control groups. These results suggested that rTgMIF protein triggered a protective effect against type 1 acute toxoplasmosis. In chronic infection with type 2 PRU bradyzoites, the data indicated that the rTgMIF protein vaccine caused a 62.26% reduction in parasite brain cysts compared to the control groups (*p* < 0.01). Therefore, these results revealed that the rTgMIF protein induced a protective effect against type 1 and 2 strains.

Cytokines are critical to promote T helper cell activation. Previous studies have shown that IFN-γ is associated with a Th1 immune response, whereas IL-4 is associated with a Th2 response (Romagnani, 1994; Abbas et al., 1996). IL-4 counteracts the secretion of IFN-γ and facilitates B cell generation, differentiation, and maturation (Chapoval et al., 2010; Bao and Cao, 2014). Most importantly, IFN-γ is a crucial mediator of immune responses against *T. gondii* during the whole period of infection (Dupont et al., 2012; Yarovinsky, 2014). In our study, IFN-γ and IL-4 were quantitatively tested to evaluate the protective effect of the vaccine. Our results revealed that 5 μg of rTgMIF evoked higher levels of IFN-γ and slight higher levels of IL-4 from immunized mice in comparison with other groups. Therefore, our findings are consistent with previous reports that mice immunized with recombinant proteins generated a mixed Th1/Th2-type immune response (Wang et al., 2014; Ching et al., 2016; Sonaimuthu et al., 2016). Meanwhile, many studies have also shown that a Th1-predominant immune response is more critical for effective protection against *T. gondii* infection (Lieberman et al., 2004; Kemball et al., 2007; Suzuki et al., 2011; Dupont et al., 2012; Sturge et al., 2013; Lopez-Yglesias et al., 2018). The rTgMIF vaccine induced high levels of IFN-γ and slight higher IL-4 levels, indicating that a bias toward the Th1-type immune response was induced by rTgMIF vaccines. As is well-known, specific IgG antibodies exert an important role in controlling *T. gondii* infection in cooperation with macrophages for resisting secondary invasion (Johnson et al., 2004; Dupont et al., 2012; Wang et al., 2013, 2017). The IgG antibody mainly inhibits the adhesion of the parasite to host cell receptors and eliminates the parasites (Zhou and Wang, 2017). Therefore, we analyzed a specific IgG antibody. The dose-dependent results indicated that anti-MIF IgG antibodies were generated at 2 weeks after preinjection with 5 μg rTgMIF. The levels of IgG antibodies from the immunized groups were higher compared with mice of the control groups (*p* < 0.0001). These findings indicated that the vaccine could trigger stronger humoral and cellular immune responses against *T. gondii* infection.

To further evaluate the protective effect of rTgMIF protein vaccines against *T. gondii* infection, the best immunization dose (5 μg rTgMIF) was utilized to perform the experiment. Prolonged survival was observed in immunized BALB/c mice, with approximately 25% of the immunized mice remaining alive at the end of the analysis period. In chronic infection challenge, the data indicated that the rTgMIF protein vaccine caused a 62.26% reduction in the number of parasite brain cysts compared to the PBS group (*p* < 0.01), which were higher than previous studies using other antigens (Hu et al., 2017; Yang et al., 2017; Zhou and Wang, 2017; Gao et al., 2018). The result suggested that rTgMIF protein could strongly induce protective effect against chronic toxoplasmosis. Together these results revealed that the rTgMIF protein provided partial but effective protection against both chronic and acute *T. gondii* infection.

## Conclusion

Our research demonstrated that rTgMIF successfully triggered a strong and specific immune response, and the vaccinated BALB/c mice had a certain protective effect against acute and chronic *T. gondii* infection. Therefore, rTgMIF should be considered to be a potential vaccine candidate against toxoplasmosis. Complete protection could be achieved by binding TgMIF to other immunogenic antigens against *T. gondii* challenge in the future.

## Author Contributions

JD and JS conceived and designed the experiments. KL, HW, LC, HC, and MW performed the experiments. KL, LC, HW, HC, MW, RA, DC, and LY analyzed the data. KL, HW, LC, MW, and JD wrote and critically revised the manuscript. All authors read and approved the final version of the manuscript.

## Conflict of Interest Statement

The authors declare that the research was conducted in the absence of any commercial or financial relationships that could be construed as a potential conflict of interest.
